# Antenatal depression and its correlates on northwestern Ethiopian women: community-based cross-sectional study

**DOI:** 10.11604/pamj.2020.36.239.19890

**Published:** 2020-08-04

**Authors:** Eyerusalem Desta Zelalem, Mengstu Melkamu Asaye, Muhabaw Shumye Mihret

**Affiliations:** 1 University of Gondar Comprehensive Specialized Hospital, Gondar, Ethiopia,; 2 Department of Women and Family Health, School of Midwifery, College of Medicine and Health Sciences, University of Gondar, Gondar, Ethiopia,; 3 Department of Clinical Midwifery, School of Midwifery, College of Medicine and Health Sciences, University of Gondar, Gondar, Ethiopia

**Keywords:** Determinants, depression, Gondar, pregnancy, prevalence

## Abstract

**Introduction:**

mental health during pregnancy is a very important but neglected problem in most African countries including Ethiopia. In general, there was a scarce of studies on antenatal depression at the community level in Ethiopia. Therefore, this study was aimed at assessing the prevalence and correlates of antenatal depression among postpartum women in Gondar city, Northwest Ethiopia.

**Methods:**

a community-based cross-sectional study was conducted in Gondar city among 526 women from July 01^st^ to 30^th^/2018. A cluster sampling technique was employed and an interviewer-administered semi-structured questionnaire was utilized to collect the data. The data were entered into Epi-info version 7.0 and exported to SPSS version 20. Both bivariate and multivariable logistic regression analyses were performed. The level of statistical significance was declared based on the AOR with 95% CI and P-value ≤0.05.

**Results:**

the prevalence of ante partum depression was 24.1% (95% CI: 20.5-27.5) and it was independently predicted by relatives’ mental illness (AOR = 2.30; 95% CI: 1.17-4.53), sex preference (AOR = 1.80; 95% CI: 1.07-3.02), lack of relatives’ support (AOR = 2.07; 95% CI: 1.12-3.87), unhappy marriage (AOR = 2.94; 95% CI: 1.81-4.76), history of depression (AOR = 5.23; 95% CI: 2.87-9.50) and no or one alive child (AOR = 1.78; 95% CI: 1.13-2.79).

**Conclusion:**

the prevalence of ante partum depression was high and connected to poor psycho-social experiences. Therefore, building-up of family's network, fortifying relatives' support, resolving unhappy spousal relationships, and assuming early screening and intervention would degrade its burden.

## Introduction

Mental health disorders (MHDs) remain the major public health problem and is predicted to be the second leading cause of global burden disability by 2020 [1]. Women become more susceptible to MHDs during pregnancy and postpartum. Fortunately, the emphasis has been paid to postpartum MHDs [2]. In contrast, antepartum MHDs abide by an overlooked public health challenge in Africa. The most prevalent mental disorder during pregnancy is antenatal depression (AND). Antenatal depression (AND) is characterized by loss of interest, lack of concentration, disturbance of thinking, the difficulty of keeping up with normal family and work responsibilities, and feel of hopelessness [3,4]. Generally, it is a syndrome of symptoms of mood swings, anxiety, loss of interest in normally enjoyable activities, and eating disorder with extreme weight changes. It can affect women’s feelings and perceptions drastically. The impact of AND can be extended to women’s entire reproductive lifetime unless timely screening and interventions are undertaken. Furthermore, AND is one of the major maternal health problems which even can lead to maternal and/or fetal death in connection to suicidal attempts directly or its desperate consequences indirectly [5]. Its impacts may not be limited to the women but also can be extended to the family and the community at large [4].

The magnitude of AND shows a regional variation in the globe. For example, its magnitude is reported to 14.2% in Brazil [2], 47% in South Africa [6], 38.8% in Tanzania [7], and 25.33% in Ethiopia [3]. Globally, compared to studies done on postpartum depression, researches conducted on AND are few in number and institutional-based in nature, which has, thus, less representativeness of the community where the real extent of the problem has existed. The existing literature showcase that the risk of AND is higher among women who have no ANC visit, work exclusively in the home, have a history of depression, encounter domestic violence, have a history of adverse pregnancy outcome, pose no live child and have unplanned pregnancy [5,8]. In Ethiopia, there is scarce of shreds of evidence on the extent and risk factors of AND. On top of that, almost all the available studies are facility based in nature. Therefore, better community representative studies are essential to estimate the real extent of the problem and to explore the corresponding correlates to overcome the mortality and morbidities which occur in connection with AND, and thereby, to achieve the Sustainable Development Goal (SDG) three [9]. Having this insight, the current study was aimed at assessing the prevalence and correlates of antenatal depression at the community level among postpartum women in Gondar city, Northwest Ethiopia, 2018.

## Methods

We conducted a community based cross-sectional study from July 01- 30/2018. The study was undertaken in Gondar city, Northwest Ethiopia. Gondar city, which is one of the historical cities in Ethiopia, is situated 189 kilometers away from Bihar-Dar - the capital city of Amhara national regional state, and 750 kilometers away from Addis Ababa - the capital city of Ethiopia. According to the 2017/18 population projection, the total population size of Gondar City is estimated to be 338,646. Out of this, 23.58% of the women are in the reproductive age group. The city has about 21 kebeles (the smallest administrative unit), one comprehensive specialized hospital, 8 health centers, three private maternity specialty clinics, and one private primary hospital. Women within six weeks postpartum and have resided in Gondar city at least six months before the study period were included in the study. The sample size was calculated using a single population proportion formula with the following assumptions: prevalence of AND- 19% [10], a margin of error -5%, design effect - 2, and non-response rate - 10%. This yields a sample size of 521. However, the total eligible women in the selected clusters were 526. Thus, we included all (526) of the participants in the study. A cluster sampling technique was employed to select the study participants. Initially, we randomly selected 8 out of 21 kebeles (clusters) in the city. Thereafter, we included all eligible women in the selected kebeles (clusters). The dependent variable for this study is ante partum depression (AND) which is dichotomized as ‘Yes’ and ‘No’. Whereas, the explanatory variables include socio-demographic variables (i.e. age, ethnic group, marital status, educational status), obstetric related variables (i.e. parity, abortion, unplanned pregnancy, pregnancy complication, stressful life event during pregnancy, and sex preference) and psycho-social related variables (i.e. history of depression before pregnancy and family history of psychiatric problem, poor husband support, domestic violence, relatives support and spouse with husband).

**Antenatal depression** is a form of clinical depression that can affect a woman during pregnancy. We measured ante-partum depression using the Edinburgh Postnatal Depression Scale (EPDS). Accordingly, women with EPDS of >13 were assigned to have ‘antenatal depression’ (i.e. labeled as ‘Yes’) while those women with EPDS of <13 were reported to have ‘no antenatal depression’ (i.e. labeled as ‘No’) [11]. Data were collected through the interviewer-administered semi-structured and pretested questionnaire. The questionnaire was prepared in English, translated into Amharic (local language), and back to English to maintain consistency of the tool. A total of 10 personnel were recruited for the data collection process. These included eight diploma midwifery students for data collection and two BSc midwifery professionals for supervision. One-day training was provided for data collectors and supervisors on the purpose of the study and techniques of data collection. The data collectors have checked each questionnaire for completeness before leaving the corresponding study participant. Daily based supervision was undertaken during the data collection period. A pretest was conducted on 5% of the sample size to check the respondent’s response, the language’s clarity, and the tool’s appropriateness. At the end of the pretest, necessary correction measures were performed. Data cleaning was performed to check for accuracy, completeness, consistencies, and missed values and variables. Data were checked, coded, entered into Epi Info version 7.0, and then exported to SPSS version 20 for analysis. Descriptive statistics were performed and the corresponding results were described in frequencies and percents, and then presented with texts and tables. The binary logistic regression model was fitted. First, bivariate logistic regression analysis was done to test the association between the outcome and each explanatory variable. Variables having a p-value of less than 0.2 in the bivariate analysis were entered into the multivariable logistic regression to control the possible confounders. Thus, both Crude and Adjusted odds ratios were computed. Finally, AOR with 95% CI and its P-value ≤0.05 was used to declare the statistically significant association between covariates and the outcome variable. Meanwhile, Model fitness was checked using the Hosmer-Lemeshow goodness of fit test.

## Results

A total of 526 informants were involved in the present study, making a response rate of 100%. About 30 (5.70%) of the participants were teenaged while 84 (15.97%) of the respondents were advance aged with the mean (± SD) age of 28.7 (±5.23) years. Nearly one-fifth (19.96%) of the participants were unmarried at the time of the interview. More than one-tenth (13.88%) of the participants had never attended formal education and about two-fifth (42.01%) of the respondents were housewives by occupation. Regarding spousal information, about 190 (45.1%) of the spouses were governmental employee, whereas, about 53 (12.6%) of them had never attended any formal education (Table 1). When the informants obstetrics condition were asked and assessed, about 163 (31%) of the participants were primiparous and 117 (22.24%) of the respondents had a history of abortion. More than a quarter (27.38% and 27.57%) of the participants experienced unplanned pregnancy and complications in the index pregnancy respectively. Nearly half (49.17%) of the respondents received incomplete ANC during the most recent pregnancy (Table 2). About 46 (8.8%) of the respondents admitted that they had relatives with mental illness and 28 (60.9%) of the mentally ill relatives were reported to be mothers for the respondents. More than one-tenth (11.98%) of the participants admitted that they had a history of depression before the index pregnancy. Whereas, nearly a quarter (24.1%) of the studied women reported that they were not happy with their husband’s relationship, and one-fifth (19.2%) of the study informants complained that they were abused by their husband during the index pregnancy. Concerning the type of abuse, substantial (71.3%) of the abused women was encountered verbal abuse (Table 3).The prevalence of antenatal depression (AND) was found to be 24.1% (95% CI: 20.5-27.8) Both bivariate and multivariable logistic regression analyses have been performed. First, all variables have been subjected to the bivariate logistic regression analysis. Accordingly, a history of family mental illness, sex preference, poor antenatal follow up, unplanned pregnancy, abuse by a spouse, poor relatives’ support, unhappy marriage, previous history of depression, low family income, and one or no alive child were associated with AND. Thereafter, the variable’s with P-value < 0.2 have been entered into the multivariable logistic regression model and the result exhibits that about six variables such as relatives’ mental illness, sex preference, no relatives’ support, unhappy marriage, depression history, and one or no alive child have a statistically significant association with AND.

**Table 1:** sociodemographic characteristics of the respondents in Gondar city, Northwest Ethiopia, 2018 (n=526)

Variables	Number	Percent
Ethnicity		
Amhara	394	74.9
Tigre	64	12.2
Kemant	65	12.3
Other*	3	0.6
**Religion**		
Orthodox	345	65.59
Muslim	152	28.90
Protestant	29	5.51
**Mothers´age (in years)**		
15-19	30	5.70
20-24	79	15.02
25-29	190	36.12
30-34	143	27.19
35-50	84	15.97
**Marital status of the mother**		
Single	59	11.22
Married	421	80.04
Divorced	40	7.60
Widowed	6	1.14
**Maternal educational status**		
Unable to read and write	14	2.66
Read and write (with no formal education)	59	11.22
Primary school	89	16.92
Secondary school	216	41.06
College and above	148	28.14
**Husband educational status (n = 421)**		
Unable to read and write	10	2.4
Read and write (with no formal education)	43	10.2
Primary school	53	12.6
Secondary school	138	32.8
College and above	177	42
**Maternal current occupation status**		
Housewife	221	42.01
Student	46	8.75
Government employee	136	25.86
Non-government employee	96	18.25
Daily laborer	27	5.13
**Current occupation of husband (n = 421)**		
Government employed	190	45.1
Non-government employed	100	23.8
Daily laborer	30	7.1
Merchant	101	24
Family income (ETB*)		
<750	24	4.56
750-1200	53	10.08
> 1200	449	85.36

* other=Gurage and Agew; ETB^*^=Ethiopian Birr

**Table 2:** obstetric related characteristics of the respondents in Gondar city, Northwest Ethiopia, 2018 (n=526)

Variables	Number	Percentage (%)
**Parity**		
1	163	31
2	173	32.9
3	110	20.9
≥4	80	15.2
**Number of live children**		
≤1	183	34.4
≥2	343	65.21
**History of abortion**		
Yes	117	22.24
No	409	77.76
**Number of abortion (n=117)**		
1	110	94.02
2	7	5.98
**Planned pregnancy**		
Yes	382	76.62
No	144	27.38
**Complication during the index pregnancy**		
Yes	145	27.57
No	381	72.43
**Type of complication during the index pregnancy (n=145)**		
Vaginal bleeding	50	34.48
Severe headache	56	38.62
Blurring of version	28	19.31
Other^*^	11	7.59
**ANC follow up during the index pregnancy**		
Yes	484	92.02
No	42	7.98
**Number of ANC visits (n=484)**		
≤3	238	49.17
≥4	246	50.83

**Table 3:** psycho-social related characteristics of the respondents in Gondar city, Northwest Ethiopia, 2018 (n=526)

Variables	Number	Percentage (%)
**Of your relatives, who is suffered from mental illness? (n=46)**		
Brother	4	8.7
Sister	14	30.4
Mother	28	60.9
**Ever had a depression history?**		
Yes	63	11.98
No	463	88.02
**Are you happy with your relationship to husband?**		
Yes	399	75.86
No	127	24.14
**What type of abuse you faced by your husband? (n=101)**		
Verbal	72	71.2
Physical	24	23.8
Verbal and physical	5	5
**Are you happy with the relationship with your husband’s families?**		
Yes	291	53.3
No	235	46.7

Thus, the odds of AND were higher among women who had a history of relative's mental illness compared with the reference group (AOR = 2.30; 95% CI: 1.17-4.53). Women who had sex preference for the index child were 1.80 folds more likely to experience AND as compared to those women who hadn’t (AOR = 1.80; 95% CI: 1.07-3.02). Similarly, women who had not got support from their relatives were 2.07 times more probably to develop AND as compared to their counterparts (AOR = 2.07; 95% CI: 1.12-3.87). Likewise, the odds of facing AND were 2.94 times higher among women having an unhappy marriage than those women with happy marriage (AOR = 2.94; 95% CI: 1.81-4.76). Also, mothers with a previous history of depression were more presumably to be positive for AND compared with those women with no history (AOR = 5.23; 95% CI: 2.87-9.50). Again, the chance of manifesting AND was higher among women who had one or no alive child than those mothers who posed at least two alive children (AOR=1.78; 95% CI: 1.13-2.79) (Table 4).

**Table 4:** correlates of antenatal depression among respondents in Gondar city, Northwest Ethiopia, 2018 (n=526)

Variables	Antenatal depression		COR (95%CI)	AOR (95% CI)
	Yes (%)	No (%)		
**Family mental illness**				
Yes	19(41.3)	27(58.7)	2.42(1.30,4.56)	2.303(1.17, 4.53)
No	108(22.5)	372(77.5)	1	1
**Sex preference**				
Yes	65(31.9)	139(68.1)	2.55(1.39, 3.57)	1.80(1.07, 3.024)
No	50(15.5)	273(84.52)	1	1
**Antenatal follow-up**				
Yes	105(21.7)	379(78.3)	1	-------------------
No	22(52.4)	20(47.6)	3.97(2.08, 7.55)	
**Planned Pregnancy**				
Yes	77(20.2)	305(79.8)	1	--------------------
No	50(34.7)	94(65.3)	2.11(1.38, 3.22)	
**Husband abuse**				
Yes	40(39.6)	61(60.1)	2.54(1.60, 4.05)	-------------------
No	87((20.5)	338(79.5)	1	
**Relatives support**				
Yes	101(21.6)	366(78.4)	1	1
No	28(47.5)	31(52.5)	3.27(1.74, 6.36)	2.07(1.12, 3.87)
**Happy mother – spouse relationship?**				
Yes	76(19.1)	323(80.9)	1	1
No	51(40.2)	76(59.8)	2.85(1.85, 4.40)	2.94(1.81, 4.76)
**Depression history**				
Yes	34(54)	29(46)	4.66(2.70, 8.05)	5.23(2.87, 9.50)
No	93(20.1)	370(79.9)	1	1
**Family income (ETB)**				
< 700	12(48)	13((52)	3.40(1.50, 7.68)	-------------------
700-1200	19((36.5)	33(63.5)	2.12(0.61, 4.22)	--------------------
>1200	96(21.4)	353(78.6)	1	
Number of alive children				
≤1	58(31.7)	125(68.3)	1.84(1.23, 2.77)	1.78(1.13, 2.79)
≥2	69(20.2)	274(79.8)	1	1

## Discussion

Antenatal depression (AND) remains an overlooked psychiatric problem in many African countries including Ethiopia. Although certain studies had been conducted on AND in Ethiopia, they were institutional-based, and thus, lacked representativeness of the community where the real extent of the problem has existed. Thus, the primary aim of this study was to estimate the prevalence and its correlates of AND at the community level among postnatal women in Northwest Ethiopia. Therefore, the findings of this study may have better representativeness of the community regarding the magnitude and the risk factors of AND compared with the previous studies. Furthermore, the findings of this study will supply important inputs to the clinical as well as the public health care interventions on proactive prevention, treatment, support, and counseling cares to target AND. This study illustrated the high prevalence of AND in the study area and was significantly associated with lack of relatives’ support, sex preferences, history of relatives’ mental illness, unhappy relationships with partners, having no or one child, and previous history of depression. The prevalence of AND in the current study area was found to be 24.1%. This means, nearly one out of four women suffered from AND. The finding is in line with the results of the studies done in Korea -22.6% [12] and Nigeria -24.5% [5]. It is also consistent with the findings of studies conducted in different parts of Ethiopia such as Addis Ababa -24.94% [10], a systematic review done in Ethiopia -25.33% [3] and Gondar University Hospital -23% [13]. The consistency in magnitude of AND across the aforesaid studies could be partially due to the shared sub-standardized socioeconomic status and poor obstetric/gynecologic characteristics of the respondents [3,5,13]. However, the prevalence of AND in the current study is higher than the results reported from other previous studies done in Washington - 9.9% [14] and Rio de Janerio - 14.2% [15]. It is also higher as compared to the findings of a systematic review on Asian women -20% [16]. The discrepancy might be attributed to variation in sample size. For instance, the numbers of study participants in the study in Washington were more than three folds (i.e. about 1,888 women) of the study participants in the current study (i.e. 526 women) [14]. Moreover, the disparity in the magnitude of depression could be explained by variation in the study population: unlike in the current study, the study population in some previous studies were pregnant women [2,14]. By logic, a lower proportion of AND could be reported if the data collected during prenatal than the postnatal period. This is because certain non-depressed pregnant women at the time of the interview could develop the condition later over the remaining course of the prenatal period. From this concept, we can recommend that cross-sectional studies which aim at assessing the magnitude and associated factors of AND ought to be conducted upon completion of a prenatal period when the courses and events of the pregnancy are entirely ended so that full courses of events and conditions will be investigated all in all despite the chance of recall bias reports. Besides, the difference in magnitude of AND might be connected to better economical and living status of the study participants in the former studies. In this insight, a population with better socio-economic status could be less susceptible to AND. This correlation further entails that emphasizing improving the social status of the women can be an important entry point to overcome AND. And this concept is under the Sustainable Development Goals (SDGs) which put the priority on poverty elimination as a very important step in creating global health and wellness [9].

On the other hand, the magnitude of AND in this study is lower than what was reported from previous studies undertaken in Pakistan - 70% [17], rural South Africa - 47% [6], Cape Town in South Africa - 39% [18] and Tanzania - 38.8% [7]. This disagreement in magnitude might be accredited to variation in sample size again: it is quite higher in the current study than the previous studies; 526 versus165 [17] and 526 versus 109 [6]. Furthermore, the dissimilarity could be ascribed to variation in the socio-demographic characteristics of the study participants. For instance, the proportion of unmarried study participants in the study done in rural South Africa is four times higher than what was reported in the current study (91.7 % versus 19.96%) [6]. Likewise, about 55.1% of the respondents in rural South Africa had no cohabitation with the baby’s father. In this regard, studies advocate that unmarried marital status and lack of baby’s father support are among the strong correlates of AND [12,19]. Also, the difference could be related to dissimilitude in study settings as some previous studies were undertaken in rural areas [6]. In the current study, the odds of antenatal depression were higher among women who had a history of relative’s mental illness, sex preference, lack of relatives’ support, unhappy relationships with the partner, self depression history, and no or one child. The current study demonstrates that the probability of developing AND is higher among women who had relatives with mental illness than their congruent. This entails that depression has hereditary bases. The association between self and relative’s mental illness can be also justified with the reality that relatives are likely to share similar lifestyles thereby to partake risk factors of depression commonly. This finding is corroborated by a previous local study done in Harare [20].

In our study, sex preference by the mother has a statistically significant association with AND. Accordingly, women who had sex preference were 1.8 times more likely to develop AND compared with the reference group. The finding is supported by previous studies [16,20]. In this perspective, existing shreds of evidence point out that multi-parous pregnant women tend to prefer the opposite gender to the previous babies' gender. Thus, women who become aware of the unwanted gender of the unborn baby are likely to feel a sense of unfortunate. Even pregnant women who are clueless about the sex of unborn babies tend to get worried about gender if they have a strong ambition on the sex of the fetus, consequently, they are likely to develop AND. The likelihood of developing AND was 2.1 times higher among women who had not got support from their relatives than those women who had support. The result is in agreement with the study done in South Africa [6] and a systematic review conducted in Ethiopia [3]. This association is more plausible as pregnant women who get social support are likely to discuss any issue freely, share the concerns to others, receive solutions from others, and perceive the sense of togetherness. These positive perceptions can assure them to develop confidence and happiness which in turn can minimize a crisis of mental/psychological instability. According to this study, AND is independently predicted by the status of relationship with the partner. Hence, women who had no happy relationships with their partners/husbands were nearly three folds more likely to develop depression during pregnancy as compared to those women with happy relationships. Linked to this concept, existing shreds of evidence show that women with bad marital communication/marital dissatisfaction are vulnerable to experience AND. By principle, women with marital dissatisfaction are prone to domestic violence. In this point of view, evidence depicts that domestic violence is one of the strongest correlates of AND [14,19]. And thus, such factors may aggravate the psychosocial stress which is a known precipitating factor of AND. Women during pregnancy need their partners' psychological, physical, and financial support by far. However, if their relationship is unhealthy during that critical period, they may miss such mandatory supports /cares and will develop extra mood instability in addition to facing physiological, hormonal and mental disturbance related to pregnancy. This concept answers why male partners’ support during pregnancy is strongly advocated and promoted as basic care. This idea is related to finding a meta-analysis study done in Ethiopia [21].

Based on the current study’s finding, the odds of developing AND were more than five times higher among women who had previous depression history than their counterparts. This suggests that AND tends to have recurred in the subsequent similar occasions. This might be explained by the actuality that most risk factors for the development of depression-like poor socioeconomic status are recurrent. This finding is under the existing literature [2,3,12,19]. The existing evidence exhibits that number of women’s children is correlated with AND [17]. The current study supports this fact. Hence, women who had no or one alive child were more likely to encounter depression during pregnancy as compared to those women who had at least two children. This finding is aligned with the result of the study conducted in Pakistan [17]. The association is convincing as women with fewer children are more likely to fear pregnancy loss which in turn can invoke AND.

## Conclusion

The prevalence of AND was found to be high. The odds of AND were higher among women who had a history of family mental illness, sex preference, lack of relatives support, unhappy relation with their spouse, history of depression, and no or one alive child. Therefore, building-up of family’s network, fortifying relatives’ support, resolving unhealthy spousal relationships, and assuming early screening and intervention would degrade the burden of AND and its adverse consequence.

### What is known about this topic

- Certain institution based primary studies were conducted in different settings of Ethiopia and respective findings on the prevalence and predictors of antenatal depression have been published;
- A systematic review and meta-analysis studies have been undertaken in Ethiopia and the corresponding results on the pooled prevalence and associated factors of antenatal depression have been disseminated.

### What this study adds

- To the best knowledge of the investigators, this study is the first in Ethiopia in being community-based. Thus, it will supply better community representative shreds of evidence on antenatal depression in Ethiopia.
